# Monitoring Acute Heart Failure Patients Using Internet-of-Things-Based Smart Monitoring System

**DOI:** 10.3390/s23104580

**Published:** 2023-05-09

**Authors:** Nouf Abdullah Almujally, Turki Aljrees, Oumaima Saidani, Muhammad Umer, Zaid Bin Faheem, Nihal Abuzinadah, Khaled Alnowaiser, Imran Ashraf

**Affiliations:** 1 Department of Information Systems, College of Computer and Information Sciences, Princess Nourah Bint Abdulrahman University, P.O. Box 84428, Riyadh 11671, Saudi Arabia; naalmujally@pnu.edu.sa (N.A.A.); ocsaidani@pnu.edu.sa (O.S.); 2 College of Computer Science and Engineering, University of Hafr Al-Batin, Hafar Al-Batin 39524, Saudi Arabia; 3 Department of Computer Science & Information Technology, The Islamia University of Bahawalpur, Bahawalpur 63100, Pakistan; umer.sabir@iub.edu.pk (M.U.); zaid_fahim@yahoo.com (Z.B.F.); 4 Faculty of Computer Science and Information Technology, King Abdulaziz University, P.O. Box 80200, Jeddah 21589, Saudi Arabia; nabuznadah@kau.edu.sa; 5 Department of Computer Engineering, College of Computer Engineering and Sciences, Prince Sattam Bin Abdulaziz University, Al-Kharj 11942, Saudi Arabia; k.alnowaiser@psau.edu.sa; 6 Department of Information and Communication Engineering, Yeungnam University, Gyeongsan 38541, Republic of Korea

**Keywords:** IoT, smart healthcare, patient mortality prediction, deep learning, heart disease

## Abstract

With technological advancements, smart health monitoring systems are gaining growing importance and popularity. Today, business trends are changing from physical infrastructure to online services. With the restrictions imposed during COVID-19, medical services have been changed. The concepts of smart homes, smart appliances, and smart medical systems have gained popularity. The Internet of Things (IoT) has revolutionized communication and data collection by incorporating smart sensors for data collection from diverse sources. In addition, it utilizes artificial intelligence (AI) approaches to control a large volume of data for better use, storing, managing, and making decisions. In this research, a health monitoring system based on AI and IoT is designed to deal with the data of heart patients. The system monitors the heart patient’s activities, which helps to inform patients about their health status. Moreover, the system can perform disease classification using machine learning models. Experimental results reveal that the proposed system can perform real-time monitoring of patients and classify diseases with higher accuracy.

## 1. Introduction

We are living in an age in which technology has revolutionized the world. The technological revolution has happened in communication, business, education, medical care, and many other areas. Digital technology removes barriers of distance, enlarges thinking, and benefits businesses. In particular, since the spread of COVID-19, the use of modern smart applications has increased massively. Such applications are used for a variety of applications, including medical care. Furthermore, the birth of the Internet is considered a major contribution to technological advancement [1]. The Internet removes barriers to digital technology and provides access everywhere. Social media is another source of information and has a large impact on the young generation. Handy technology provides instant data on social media.

The Internet of Things (IoT) is an emerging field of computer science that has impacted the world in a short period of time. The concepts of smart homes [2], smart-resource-based cities [3], smart driving [4], and smart farming [5] have changed the living and working styles of many people around the world. Smart devices are embedded in smart homes and cities, and things are controlled by smart devices. In the near future, real-time human activities will be monitored by smart devices, and real-time data will be collected by tagging sensors within the human body [6]. Human health can be monitored in real-time by sending the information obtained using the sensors to medical consultants. The Internet of medical things (IoMT) is the field in which health equipment is connected with IoT [7]. Authors have discussed IoMt using wearable devices and AI [8]. This concept gives new directions to the health field and opens up new doors of development. As in the medical field, accurate and timely diagnosis and intervention are the main factors in health-related issues, so substantial work is required to fulfill this gap and develop intelligent systems for health monitoring.

**Figure 1:**
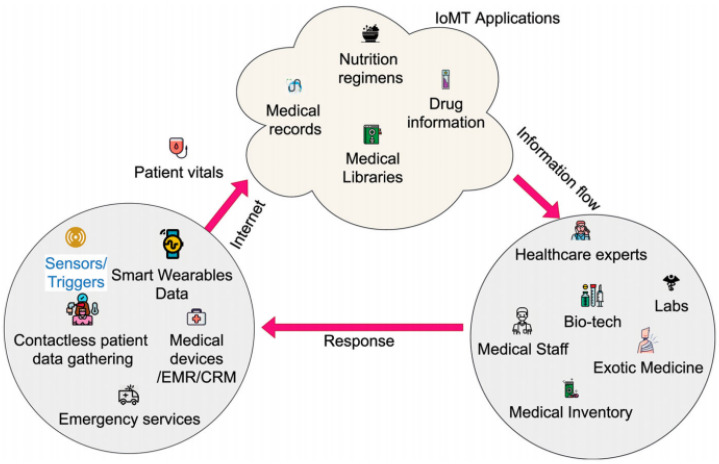
IoMT mechanism [14].

With the development of information technology (IT), medical fields are being revolutionized in developed countries. In a traditional medical system, huge crowds, power consumption, and routine work all burden the system and lead to delays in facilitation. In IoMT, wearable sensor devices are connected to the care provider’s smart devices, and they can monitor the real-time patient health record and treat the patient accordingly [9]. IoMT provides a low-cost and quick solution by remotely monitoring the patient’s health. With the spread and rise of chronic diseases, the medical systems of underdeveloped countries face significant issues in managing large numbers of patients due to a shortage of staffing and resources. In the medical field, quick responses significantly impact saving lives. Figure 1 shows the IoMT mechanism in the physical environment.

Artificial intelligence (AI) is a domain of computer science that induces intelligence in machines and makes smart devices capable of working without human intervention. Smart devices make smooth connectivity using AI and work innovatively. AI processes help to find hidden patterns in the large volume of data received from smart devices [10]. Moreover, the AI process also makes recommendations to improve the performance of the systems. The domains of AI and machine learning (ML) greatly help in solving today’s complex problems. In every field of life, computational systems are designed using AI and ML to solve significant dynamic problems. Combining IoT, AI, and ML can change how people live and interact in their daily lives. In the medical field, large datasets are gathered using smart devices and sensors. AI and ML algorithms are applied to find underlying hidden patterns to diagnose different diseases.

Using AI-based solutions, it has become easy for medical staff to work on large datasets and provide future recommendations to prevent diseases. The artificial intelligence of medical things (AIoMT) combines AI approaches with health diagnosis approaches to help in the medical field. The idea behind AIoMT is to prevent unnecessary stays in the hospital and avoid the associated health charges [11]. With the growth in the population, traditional methods of medical diagnosis need to fulfill the demand of the growing community. Because of the limited resources considering the increasing population growth, finding solutions for the efficient management of these resources is a high priority.

After COVID-19, a remote healthcare system is needed for the real-time diagnosis of various diseases. To improve healthcare facilities, it is the responsibility of academia and industry to make combined efforts to overcome these issues. Some efforts have been made in the recent past to design a smart bed concept and smart point-of-care (PoC) devices, but due to population growth, further efforts are needed. According to the world health organization (WHO), 17.9 million deaths are recorded yearly because of cardiac diseases [12]. Heart disease is a major challenge in the medical field. Heart disease arises due to the narrowing of arteries. Heart failure is the primary cause of death in most underdeveloped countries due to inadequate instant response. Old-aged people are the predominant group of heart patients; however, young patients might also be victims of this disease. In the US, one of every nine deaths are caused by heart disease [13]. Chest pain and fatigue are the primary symptoms of this disease, but it happens even without such symptoms. There is a need to develop a remote health monitoring system for heart patient that continuously monitors the patient’s activity. In the case of an emergency, remote health-monitoring systems can require an instant response. IoMT provides a lot of success in medicine, especially in rural areas where providing instant help is challenging. Early disease detection and medication can save a lot of lives. Remote health monitoring also reduces the cost of routine diagnosis due to eliminating the traveling cost and other medical overhead.

The IoMT is a multifaceted domain of IoT and medical technique and faces many challenges. Medicine is a sensitive field where the survival of human beings is essential. Therefore, a remote monitoring system in IoMT with AI is needed. The software systems in medical areas must be appropriately and extensively verified to meet medical standards. The IoMT-induced AI system must satisfy the following criteria:

**Security** The AI-inspired remote monitoring system of cardiac disease must be secure. It must not be vulnerable to adversarial attacks by other devices. The exact measurements it provides are the main advantage of these systems.
**Reliablity** The reliability of medical software systems must be achieved. A non-reliable system cannot be used in any field, particularly when the privacy and confidentiality of patients are concerned. Internal implementation issues or external problems should be ensured regarding accurate measurements. An auto-correction algorithm must be implemented in every system to avoid damage.
**Safety** The remote monitoring software must be economical and not affect the environment. The system must be human-friendly so that there is no negative impact on human lives. The medical operator, user, and designer should be able to interact with the system without harm.

In this study, a remote monitoring software system is designed using IoMT and AI approaches. This remote system takes real-time human data and processes those data in the presence of bio-medical experts. AI approaches find hidden patterns and classify cardiac patients. The remote monitoring software system obtains real-time patient data and detects heart disease. The system is helpful in early diagnosis and removes the physical barriers to reaching the hospital. The performance of the proposed system is comparable to existing systems in the literature. This study makes the following contributions:

- A brief overview of existing literature on IoMT is presented along with the major applications of IoT in medical care. In addition, major challenges in IoMT are identified.
- An IoT-based smart healthcare monitoring system is presented that collects and processes the data for heart patients. Machine learning models are incorporated to detect patients with acute heart failure.
- The performance of the proposed system is evaluated using several experiments, and a performance analysis is performed in comparison to existing state-of-the-art methods.

The rest of the paper is organized as follows: Section 2 discusses previous literature. Section 3 provides the working of IoMT. Section 4 describes the dataset used in this work and provides the scenario of the proposed smart framework. It also explains the various algorithms used in this research. Section 5 explains experimental details and evaluation parameters. A conclusion is given in Section 6.

## 2. Related Work

In this modern age, multiple technologies have been developed to monitor medical data. With the invention of sensors, the development has been taking place at a rapid pace. Sensors are used for real-time monitoring and data collection. Smart devices are full of sensors and are used for data collection. In [15], a technique based on a ring sensor is used to monitor the patient. A wearable ring containing the sensor is used for real-time monitoring of cardiac patients. In [16], a technique based on an ear sensor is used for continuous monitoring of heart patients. These sensors are small in size and wearable among persons of different ages alike which makes the monitoring process easy.

Heart patients need an instant response in case of an emergency. Most fatalities happen due to delays in early diagnosis and inefficient methods. In [17], the authors designed a technique to take advantage of IoT technology using cloud sources for early diagnosis. One of the biggest challenges in medical fields is maintaining data. Clouds have huge volumes of storage capacity, so combining IoT technology with the cloud can have a huge impact. The study [18] designed an energy-efficient protocol for healthcare applications using dynamic channel coding by combining physical and multiple access layers. The aim is to optimize energy usage and maintain a lifetime of nodes in a network.

During the COVID-19 pandemic, the health of front-end medical staff became vulnerable to this disease, and extra care was required to deal with patients. Consequently, remote health monitoring applications helped medical staff to diagnose patients effectively. Such systems are realized using IoT devices [19,20]. In addition, image processing applications are installed at different public points for real-time surveillance of the public. The data from such installments can be obtained via IoMT by medical staff for further analysis [21].

Due to the advantages offered by IoMT technology, several approaches and systems have been presented recently using IoT connectors (Table 1). Jain et al. [22] proposed a health-monitoring system based on near-infrared spectroscopy and ML to analyze the glucose level. Today, IoMT edge devices are frequently used to monitor human health. It is a positive trend to help humans achieve fast-tracking and accurate results. The error ratio of the proposed glucose level detector is minimized as compared to methods available in the literature. Shui-Hua Wang et al. [23] proposed a method to classify diseases such as COVID-19, pulmonary diseases, tuberculosis, and pneumonia. The suggested method helps medical staff to diagnose diseases more accurately. This method shows better results in detecting various diseases. The authors designed an approach for remote-controlled ambulance service to improve healthcare in [24]. AI approaches are used to obtain real-time results. AI approaches are helpful in real-time applications, especially in medical applications where a fast and accurate response is needed. Similarly, Harshal Arbat et al. [25] used an approach to monitor heart patients by managing a smart health band. The band is used to measure the heart rate data, which are used in analyzing the patient’s health.

Smart devices are used today to gather health-related data to analyze human health. The study [34] designed an approach to monitor human health using a mobile application. Mobile applications can be merged with IoT and cloud computing to boost the limits of the health monitoring system. Cloud technology has enough storage to store large volumes of IoT data and provide processing services. This approach also covers the brain signal to measure the stress of the human body. Michael Fischer et al. [35] proposed a technique for non-professionals to know about various diseases. Instructions are given to the bot, which help to diagnose the patient. The integration of the bot with smart devices helps in providing better services. The accuracy of the technique is low compared to other techniques, yet it is a remarkable step toward automated disease diagnosis. The complete summary of IoT-based works is shown in Table 2.

Along the same lines, Reference [41] developed a system consisting of cloud, IoT sensors, and IoMT devices to deal with cardiac patients. The system measures the patient’s eye movement, body temperature, and oxygen level for heart disease. Similarly, authors designed a model to monitor heart disease in [42]. The proposed approach utilizes sensor data and an ensemble model in a fog environment. The model performs early diagnosis of heart patients. In [43], an Adaptive Neuro-Fuzzy inference system with multiple kernel learning is used to identify cardiac disease. The system provides better results, although the computational complexity is high. An automated approach is designed in [44] to distinguish between people at high risk of heart failure and those at low risk. The authors used the classification and regression tree (CART) and achieved specificity and sensitivity values of 63% and 93%, respectively. The existing state-of-the-art studies on cardiac disease is shown in Table 3.

## 3. How IoMT Works

This section presents technologies, challenges, the significance of health-monitoring systems, and the benefits of using IoT in healthcare.

### 3.1. Technologies

IoMT systems function at the primary level, which combines many technologies. A few of these are discussed here.

#### 3.1.1. Ethernet

Ethernet is a type of wire used in computer networking. It enables the sensor devices to transfer data via cable wires. It is also used in IoMT for connecting IoT devices and monitoring patient data in real time. Ethernet connection is reliable, high-speed, and secure. The normal speed of Ethernet is 10 Mbps.

#### 3.1.2. Bluetooth

A technical standard called Bluetooth makes it possible for electrical appliances to communicate wirelessly across short distances. Bluetooth uses short-range radio frequencies. Bluetooth is connected to other Bluetooth-enabled devices for short-range communication. The Bluetooth device mostly works around a 10 m distance. It is good for devices within the medical field.

#### 3.1.3. Zigbee

In order to reach devices farther away, Zigbee devices use a mesh network of intermediary devices to relay data across long distances. Zigbee technology is much faster than Bluetooth technology for data communication. Nowadays, ZigBee technology is mostly used with IoT technology to enhance the benefits of IoT. It is also used in medical services to deliver data on time and accurately. It uses 128-bit encryption for the security of transmission.

#### 3.1.4. Wi-Fi

In a Wi-Fi environment, computers, tablets, cellphones, and other devices may all be connected to the internet using Wi-Fi devices. Doing so establishes a network by enabling information interchange between these devices and several others. The communication range of Wi-Fi is broad, and it follows IEEE 802.11 standards. In order to enable more devices to connect to the network from farther away, an access point expands the bandwidth flowing from a router.

#### 3.1.5. Near Field Communication

For wireless connectivity within a short range, near-field communication (NFC) is used with a 4 cm range. With NFC, data can be transmitted swiftly and effortlessly across devices with one touch. To transfer data between two NFC-enabled devices, they must be close to each other and must be paired. There are two modes of operation in NFC technology, active and passive mode. In active mode, there is no need for pairing between devices, while in passive mode it needs pairing.

#### 3.1.6. Satellite

A satellite is a communications system that can pick up signals from the Earth and retransmit them using a transmitter and receiver. Satellite is positioned high above the Earth’s atmosphere. Depending on the use, satellites circle the planet in a variety of ways. Nowadays, satellites are used in weather forecasting, telecommunication, and geo-positioning. Satellites are used to analyze the region where normal access is impossible; in the near past, satellites sent images of the solar system.

### 3.2. Challenges of IoMT

As the medical field is sensitive due to the confidentiality and privacy of patient data, the security and accuracy of the medical equipment are necessary [14]. In IoMT, wireless technology is mostly used, and the privacy of the data is a great concern. A few of the most critical challenges are discussed here.

#### 3.2.1. Data Privacy

Ensuring sufficient cyber safety for health monitoring systems is one of the key issues for IoMT implementation. Security of the vast amount of private health information that is moved between systems is a problem that has to be solved [49]. Blockchain technology is used by “CoviChain” to address security and privacy concerns and prevent the exposure of personal information while gaining greater data storage capacity [50].

#### 3.2.2. Interoperability

The extent of usage is limited by the heterogeneity of devices and data from various sources, mostly due to inter-operator variation. The difficulty of data interchange between several IoMT systems with disparate capabilities makes interoperability challenging [51]. Therefore, the creation of standardized interfaces is essential, especially for programmers that facilitate cross-organizational communication.

#### 3.2.3. Cost Effectiveness

Health-monitoring systems have a significant number of sensors and medical equipment that are linked. These are expensive to upgrade and maintain, which affects both the manufacturer and the end user. Incorporating inexpensive, low-maintenance sensors can encourage the creation of more IoMT devices and help them become more widely used [52].

#### 3.2.4. Power Consumption

Another obstacle to the widespread deployment of IoMT devices is power consumption. When a sensor is attached, the majority of IoMT devices require either a regular backup solution or the usage of a strong battery [53]. Designing sustainable medical equipment that can provide its own electricity and integrating the IoMT system with renewable power sources that can also help to mitigate the global energy problem should be the current emphases.

#### 3.2.5. Environmental Impact

Biomedical sensors are created by combining a number of semiconductors composed of hazardous chemicals and rare earth elements that may have a negative environmental impact [54]. Consequently, government entities are overseeing and supervising the production of sensors.

### 3.3. Significance of IoT-Based Healthcare-Monitoring Systems

Researchers and medical experts are paying close attention to the development of monitoring systems for healthcare. Numerous successful research initiatives in this field have been taken, and many more are now underway [55]. The gap between people treated by healthcare professionals and those in need of treatment is constantly increasing, owing to the fast-expanding population of elderly persons and patients with chronic conditions. The main disadvantage is that healthcare is only available in hospitals; as a result, it is unsuitable for the elderly and impaired persons, and it cannot always satisfy their needs [56]. The IoT, through sensor values and telecommunications, provides an effective and practical answer to the issue of real-time monitoring of the health state of the elderly. It has been demonstrated that the IoT, in conjunction with smart technology, may deliver a variety of upgraded and expanded services. Researchers have created a variety of emergency systems employing sensors and technology that enable intelligent and distant wireless communication. These technologies have been employed in a variety of medical applications, most notably in monitoring the health of elderly people. By recording crucial vital signs, data on general health and risky circumstances may be acquired [57].

### 3.4. Benefits of Using IoT in Healthcare

The IoT is expected to have a significant impact on how healthcare is provided. We have entered a completely new era in terms of the interaction between applications, technologies, and people providing healthcare solutions. The ability to create an integrated healthcare network with the help of the IoT has dramatically improved, giving new insights and tools. Healthcare processes that formerly needed a lot of time and were error-prone due to human involvement can now be automated thanks to IoT and AI. For instance, a lot of hospitals now employ networked equipment to regulate temperature and ventilation in operating rooms. There are several ways that IoT may advance healthcare, but the following are some of the more significant advantages:

- Reduced probability of human errors,
- Resolution of restrictions regarding distance and physical visits,
- Less paperwork and record-keeping,
- Early detection of chronic disorders,
- Improved handling of medication,
- Immediate medical attention,
- Improved results of therapy.

## 4. Materials and Methods

### 4.1. Smart Healthcare Framework

**Figure 2:**
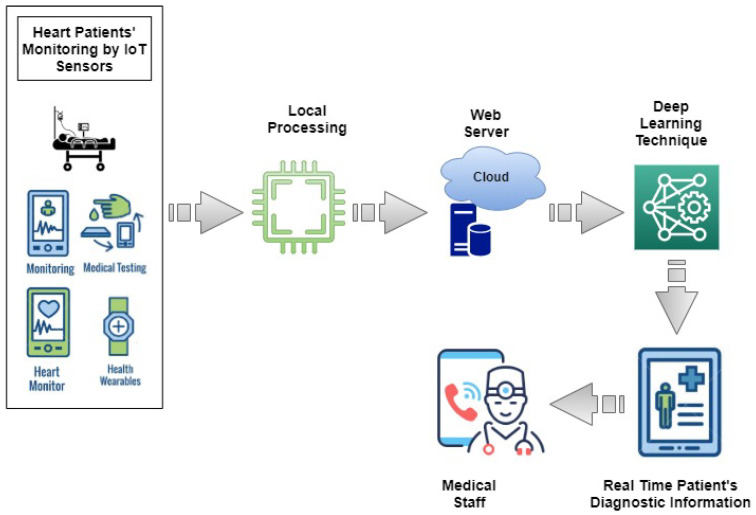
Workflow of the proposed monitoring framework.

An IoT-based healthcare platform for people with heart failure is presented in this section. The proposed framework is based on cloud services, AI and IoT. Figure 2 shows the suggested system’s design for smart healthcare. With the help of IoT devices, this framework makes it easier for medical personnel to keep track of their patients’ health. IoT and cloud-based technologies allow medical professionals to access the medical records of heart failure patients at any time and from any location. The complete flow of the proposed architecture is shown in Algorithm 1.
**Algorithm 1** Steps of the Proposed IOT Architecture  1: **Read:** Medical healthcare labs, prescriptions, and patient history records.  2: **Connect:** Make a Firebase data connection.  3: **Authentication:** Healthcare verification.  4: **if** Transmission == 1 **then**  5:  Transmitting healthcare all data by JSON file  6:  Medical analysis data are used to make predictions by deep learning method.  7:  These predictions are used to generate the patient report.  8:  Move medical report from Firebase to officer device.  9: **else**10:  Medical healthcare data is saved in the local storage of the device.11:  Local storage data are moved to Firebase when the connection is successful.12: **end if**

In this IoT-based smart healthcare system, the security factor is added using Zigbee and Firebase IoT authentication. When medical officers send patients’ data to cloud storage, 128 bits of security encryption are added to the JSON file as a token. The Firebase cloud function validates the officer’s device token by generating a custom token with the officer’s correct credentials details and custom token claims. The device-generated 128 bits token and Firebase custom token is considered proof of identity for all real-time exchange of data between the two users. After authentication, the authorization process is performed using Firebase’s general Security rules. This three-step-based security mechanism of Firebase can be summarized as follows.

- The device token proves that the request came from an authorized device, but it has no useful information. Firebase servers would not be able to easily determine who is the owner of a device token.
- A custom token contains the user identity but lacks their profile information. In addition, this token cannot be implicitly trusted by Firebase servers, since the service account used by our Cloud Function is not guaranteed to be authorized. For example, we might decide to revoke it or rotate its key.
- The claims from a custom token are validated by the signInWithCustomToken API. Then, the backend generates a Firebase id token. This token contains the user profile and is irrevocable proof that its bearer is authorized to do the operations on behalf of the user. Since it cannot be revoked, it is only valid for 1 h.

Figure 2 illustrates how smart systems enable IoT to send and update patient data. The chosen equipment depends on the hospital and patient care facility. The suggested architecture examines patient data that are available in real time and enables patients to quickly obtain emergency medical care. Because these data are stored in the cloud, medical personnel may access them remotely and offer guidance based on the patient’s condition.

The major goals of the suggested smart healthcare framework are to increase the chances of survival for critically ill patients and provide heart patients with simple, affordable, and reliable monitoring. The suggested system gathers data and sends them to the cloud to be processed further using machine learning and deep learning models. Medical professionals are provided with organized data for in-depth investigation.

### 4.2. Dataset

This section discusses the dataset that was obtained from the UCI-ML repository and is based on clinical records of heart failure [58]. The dataset contains patient records for acute heart failure. There are 11 clinical characteristics that are included in the data that are gathered throughout the follow-up period. From a total of 299 dataset records, 194 belongs to male and 105 belongs to female patients. The dataset characteristics are described in Table 4.

### 4.3. Deep Learning Model

An expanding area of research in the field of artificial intelligence is deep learning. The modeling of data in deep learning gives promising results. The adoption of an automated process by medical professionals has been shown to be a highly useful and successful tool for disease diagnosis. Deep learning is a common method for processing enormous amounts of data. It eliminates the need for manual feature extraction and is being employed widely in medical data analysis.

#### Multilayer Perceptron Neural Network

When we are talking about training sets that are not large, easy implementation, speed, and the quick-result Multi-Layer Perceptron are the best choice [12]. The internal structure of the MLP comprises three layers, input, output, and hidden layers. The hidden layer is an intermediate layer connecting the input layer with the output layer during neuron processing. The internal workings of MLP are simply based on the multiplication of input neurons with weights $w_{ij}$, and the output $y_{j}$ is the sum. Mathematically, it is computed as

$$y_{j}=f\left(\sum w_{ij}*O_{i}\right),$$

In this equation, the gradient descent algorithm is assigned weights *w*, and *O* represents hidden layers.

### 4.4. RNN

When we are talking about sequential neural networks, the Recurrent Neural Network (RNN) is the best choice [12]. During processing, the input sequence of one neuron is fed to other neurons in the same weighted sequence of words in a sentence. RNN sequences are designed in a manner that generates the sequence and predicts the next word coming in the loop.

#### Convolutional Neural Network

**Figure 3:**
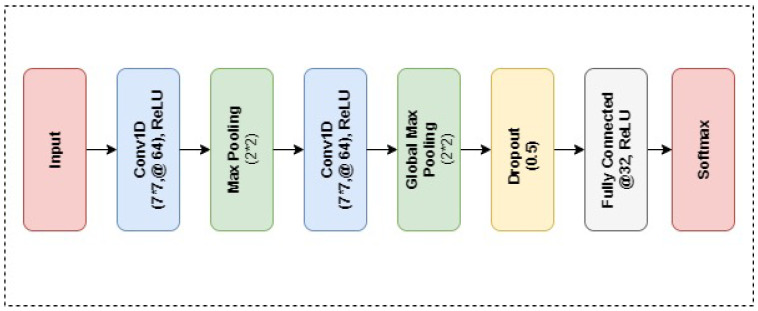
Layered architecture of the proposed CNN model.

CNN is an effective neural network model that can learn complex relations among different data attributes. A CNN is a deep learning model that can analyze the input image, rank various features and objects within the image, and distinguish between them. CNN is made of a hidden layer, node layer, input, and output layer. To obtain better results, this study uses a customized CNN architecture, as shown in Figure 3.

The proposed 8-layer architecture includes 2 dense layers, 2 max-pooling layers, and 2 convolution layers. For classification purposes in the medical field, CNN performance is the best and most accurate. In the CNN model, the Sigmoid is used as the error function and it is a backpropagation algorithm. CNN has been used for the classification of multiple diseases i.e., brain tumors, lung disease, and cardiac disease. Nowadays, it is extensively used in the medical field and deals with large amounts of data. The pooling layer in CNN can be maximum and average pooling, maximum pooling is mostly used for sharp feature extraction, while the average is used for flat feature extraction.

### 4.5. Long Short Term Memory

An improved RNN called LSTM is more operative for long-term sequences. LSTM overcame the vanishing gradient issue that RNN faces. It outperforms RNN and can memorize certain patterns. The input gate, output gate, and forget gate are the three gates that make up an LSTM. The word sequence is shown in Equations (1)–(3).

(1) $$i_{t}=\sigma \left(x_{t}U^{i}+h_{t-1}W^{i}+b_{i}\right)$$

(2) $$o_{t}=\sigma \left(x_{t}U^{o}+h_{t-1}W^{o}+b_{o}\right)$$

(3) $$f_{t}=\sigma \left(x_{t}U^{f}+h_{t-1}W^{f}+b_{f}\right),$$

where $x_{t}$ is the input sequence, $h_{t-1}$ is the preceding hidden state at current step *t*, $i_{t}$ is the input gate, $o_{t}$ is the output gate, and $f_{t}$ is the forget gate.

### 4.6. Experimental Design

**Figure 4:**
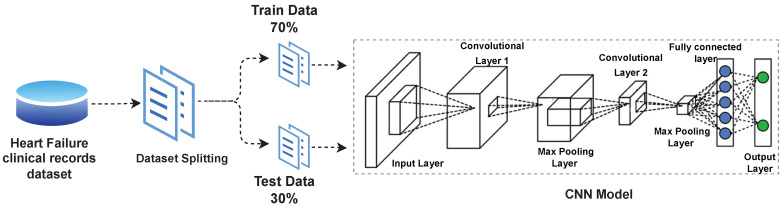
Architecture of deep learning CNN model.

In this research, an efficient health monitoring model is proposed to monitor heart patients. The model is based on a deep CNN as shown in Figure 4. In addition, LSTM, RNN, and multilayer perceptron (MLP) are also employed. The MLP model consists of an input layer, output layer, and hidden layer. The hidden layer is the processing layer and connects the input and output layers. The sum of input values with respect to weight and output is shown in Equation.

(4) $$y_{j}=f\left(\sum w_{ij}*O_{i}\right),$$

where *O* is the hidden layer and *w* is the weight value.

Experimental results are calculated on multiple-layer structures, but hidden-layer structures give suitable outcomes. Features of a heart failure dataset are provided as input to the hidden layer structure. The parametric values can be set such that the number of epochs is 25, the learning rate is 0.01, the batch size is 256, and the dropout is equal to 0.2. Another performance evaluation technique has been implemented based on the CNN approach to test its performance. A total of 8 layers are included in the CNN-based approach. The initial parametric values of the CNN-based approach are presented in Table 5.

The heart failure dataset is provided as the input in the first stage. Thirteen features of the heart failure dataset are used to train the data to provide more accurate predictions. Acute heart failure patients need early diagnosis and proper treatment to increase patients’ survival rate.

### 4.7. Evaluation Measures

In order to evaluate the accuracy of the proposed model, performance measures are calculated including precision, accuracy, F1score, etc. The accuracy of the model is important to determine, especially in the medical field, where an accurate prediction is desirable.

(5) $$Accuracy=\frac{\mathrm{Number}\;\mathrm{of}\;\mathrm{correctly}\;\mathrm{classified}\;\mathrm{predictions}}{Total\;predictions}$$

(6) $$Precision=\frac{TP}{TP+FP}$$

(7) $$Recall=\frac{TP}{TP+FN}$$

(8) $$F1-score=2\times \frac{precision\times recall}{precision+recall}$$

where TP, TN, FP, and FN represent true positive, true negative, false positive, and false negative, respectively.

## 5. Results and Discussion

The performance of the CNN-based model was evaluated against the dataset of heart patients. The deep learning algorithms MLP, CNN, LSTM, and RNN were used to predict heart failure patients and compare with the machine learning algorithms. A training-to-testing ratio of 70% to 30% was set for all models to measure the performance of the models. The Python libraries Keras and TensorFlow were used to implement deep learning algorithms. The hardware and software specifications are shown in Table 6. The system took approximately one hour time to train the data and give the final results.

Table 7 shows the performance analysis of the deep learning models regarding the accuracy, precision, recall, and F1 scores. The CNN model leads, with an accuracy score of 0.9398 and precision, recall, and F1 score values of 0.95 each.

The performance of the CNN model is better than the other deep learning models. Its performance was compared to two other models from study [59]. Reference [59] performed experiments using different scenarios, including one involving the use of oversampling by the synthetic minority oversampling technique (SMOTE). The SMOTE is utilized with CNN and Extra Tree Classifier (ETC) models. Results comparison given in Table 7 indicates that the performance of the proposed CNN model is better than both the CNN and ETC, which is employed with SMOTE in [59].

Table 8 shows the accuracy with other models employed in [59], including Decision Tree (DT), Logistic Regression (LR), Stochastic Gradient classifier (SGD), etc. All these models are utilized with the SMOTE oversampling approach. Performance comparison indicates that the proposed CNN deep learning model shows better results than all the models used in [59]. Despite the use of SMOTE in [59], the CNN model performs better than these approaches.

### 5.1. Comparison with Deep Transfer Learning Models

In addition to machine and deep learning models, a performance comparison was also carried out using transfer learning approaches. Visual Geometry Group (VGG-16) and AlexNet are the two deep neural networks employed in this study. The VGG-16 is based on convolution, connected, pooling, and padding layers, while AlexNet is also based on CNN and has millions of parameters. Table 9 shows a comparison of the performances of CNN, VGG-16, and AlexNet. The CNN model shows better performance, but precision, recall, and F1 scores of VGG-16 are also good.

Table 10 shows the comparison of time needed for training the transfer learning models and the proposed CNN model to analyze their computational complexity. The training time of the proposed model is less than those of transfer learning models, which shows its efficiency in terms of time and accuracy.

### 5.2. Results of Proposed Model for K-Fold Cross-Validation

To validate the proposed model, 10-fold cross-validation was applied. The heart failure dataset is used to validate the model. The CNN-based proposed model classifies the patient data with an average accuracy of 0.9462, while the precision, recall, and F1 scores are 0.9398, 0.9565, and 0.9481, respectively. Table 11 shows 10-fold cross-validation results of the CNN.

### 5.3. Discussion

IoT-based smart patient monitoring systems are receiving attention, especially in the context of dealing with a large number of patients and those with acute heart failure, where continuous monitoring is needed. This study presents an IoT-based monitoring system along with a deep CNN model for heart failure detection. Experiments are performed to analyze the effectiveness of the proposed CNN within the context of other machine learning, deep learning, and transfer learning models such as VGG-16 and AlexNet. Experimental results show the best results from the proposed CNN model compared to other employed models. The classification accuracy of the CNN model is 0.9368, which is better than other models.

The proposed model outperforms all machine learning and deep learning models. The accuracy, precision, recall, and F1 score performance of the proposed model is high. In order to validate the proposed CNN, 10-fold cross-validation is applied, which also shows its efficacy. Moreover, a comparison of the proposed approach is carried out with existing works regarding different features such as the number of features used, the uses of AI approaches, and IoT and patient monthly record-keeping, and results are presented in Table 12. These comparisons indicate that the proposed system is better than the existing ones.

### 5.4. Comparison with Existing Studies

The performance of the proposed models is further compared with existing studies that utilized the same dataset for experiments. Table 13 shows the comparison of the accuracy of the proposed CNN with [68,69,70,71]. The study [68] employed an optimized logistic regression for heart disease detection and obtained a 0.85 accuracy score. On the other hand, both [69,71] made use of the Naïve Bayes model and obtained accuracy scores of 0.74 and 0.86, respectively. The authors used a K-NN model in [70] for the same purpose and obtained a better accuracy of 0.92. In comparison, the proposed model obtained an accuracy score of 0.9398 and proves to be better than these studies.

### 5.5. Performance of Proposed Approach Using Real-Time Dataset

Additional experiments are performed to analyze the performance of the proposed approach using a real-time collected dataset.

#### 5.5.1. Dataset Description

This study employs the Public Health Dataset, which comprises four datasets, Cleve-Land, Hungary, Switzerland, and Long Beach V. The dataset contains 76 features; however, only 14 features are used in all published research that used this dataset.

Clinical HD data from 303 patients at CCF in Cleveland, Ohio, and across the US were collected in the dataset. The Heart Disease Database UCI_MLRepository contains this dataset and is publicly available [72]. In each of the 303 clinical cases, there are 76 attributes, as well as, a target attribute. An integer from 0 to 4 indicates the state of the patient, 0 indicates a heart patient, and [1, 2, 3] indicates a healthy subject. For the current study, binary classification is used, so the target values are set to 0 and 1 for heart patients and healthy subjects, respectively. The 282 clinical sessions include 125 instances of cardiac disease (44.33%) and 157 cases of lacking cardiac disease (55.67%). Table 14 displays features, attribute names, and domains.

#### 5.5.2. Experimental Results

Table 15 shows the results of deep learning models using the real-time dataset. Deep learning models are used because they show better performance compared to machine learning models. The performance of the proposed CNN model is better than that of other deep learning models. The CNN shows an accuracy score of 0.9534, which is better than MLP, RNN, and LSTM. It is followed by RNN, which has a 0.9449 accuracy score. The proposed CNN has better performance on metrics such as precision and F1 score and obtains the highest recall score at 0.97.

### 5.6. Comparison with Existing State-of-the-Art Approaches

The proposed health monitoring technique is compared with the state-of-the-art methods. The study [73] designed an IoT-based cardiovascular risk prediction using ensemble techniques. The ensemble techniques increase complexity and require higher computation resources, which is not appropriate for health-related systems. Moreover, these techniques generate over-fitting problems if not properly implemented. Table 16 shows a performance comparison of the proposed approach with [73]. The results show that the proposed approach has better performance.

### 5.7. Limitations and Future Directions

The main limitation of this study is that the proposed system collects data from different sources and sends them to the cloud for further analysis. The IoT-based system can be further expanded with different types of wearable medical healthcare devices that can be operated on smart devices such as smartphones, digital assistants, or tablets, which are common among medical workers. These devices provide local data storage and have only fundamental processing capabilities. The security of such devices is also low, which can compromise the confidentiality and privacy of patients’ data. Wearable and implanted IoT devices can provide continuous monitoring of patients and allow for theearly diagnosis of possible health issues.

## 6. Conclusions

A remote health monitoring system is designed in this study to monitor the health of acute heart failure. IoT technology is used to design health monitoring systems in order to access patients’ records without a physical appearance in medical clinics. The proposed smart healthcare framework is used to improve the odds of survival for critically ill patients and to provide easy, economical, and dependable monitoring for cardiac patients. The proposed system collects data and delivers them to the cloud, where they are further analyzed.

In addition, an optimized CNN model is presented for the accurate detection of heart patients, and its performance is analyzed against machine learning, deep learning, and transfer learning approaches. The experimental results indicate that the proposed models achieve better results than all the employed models with a 0.9398 accuracy score. Its performance is further validated using 10-fold cross-validation and performance comparison with existing studies using the same dataset for experiments; both prove the superior results from the proposed model. The accuracy and training time of the proposed technique are also better than those of the other models.

## Figures and Tables

**Table 1:** IOT connectors and their applications.

Ref.	Standards	Purpose
[26]	Bluetooth	Monitoring and detection of heart sounds
[27]	Bluetooth	Fitness health monitoring when engaging in physical activity
[28]	Wifi	Remote monitoring of health condition
[29]	3G	Provide fast data communication during emergencies
[30]	Satellites	Track patients and approach them in an emergency
[31]	NFC	Home surveillance for less-skilled individuals
[32]	2G, 3G	Skincare in real-time
[33]	RFID	Locating and tracking medical equipment quickly

**Table 2:** Summary of IoT-based works.

Ref.	Year	IoMT
[34]	2016	Cloud-based remote ECG system
[36]	2016	Secure healthcare system for patient
[37]	2016	Intelligent healthcare system for patients in medicine
[38]	2016	IoT-based kidney anomaly recognition system
[39]	2016	IoT enabled healthcare
[40]	2016	IoT-based mobile medical health care system

**Table 3:** Existing studies on cardiac disease.

Ref.	Methods	Data	Limitations
[43]	Neuro-Fuzzy Inference and Multi-Kernel Learning	KEGG Metabolic Dataset	Evaluating parameters are not comparable.
[45]	CNN, BiGRU, BiLSTM Ensemble	Cardiac Disease Dataset	The dataset used in this approach is not a standard dataset
[46]	Gradient Boosting, XG Boost, Random Forest Ensemble	Hungarian, Cleveland, Z-Alizadeh Sani Dataset	The complexity and price of the model are increased by stacking three models.
[47]	Generalized Discriminant Analysis and Fisher Method	SR-CAD and NSR-CAD	There is lack of training on large datasets. Training on heart rate variability needs to improve.
[48]	Random Forest, KNN, Decision Tree, Naïve Bayes	Hungary, Cleveland, VA Long Beach, Switzerland Datasets	There should be more experimentation with model combinations and feature choices.

**Table 4:** Dataset specification.

Sr. No.	Attributes	Description	Range	Measured in
1	Time	Development age	4–285	Days
2	Events	During development period patient died	0,1	Boolean
3	Gender	Man or woman	0,1	Binary
4	Smoking	Smoke addicted patient	0,1	Boolean
5	Diabetics	Diabetic patient	0,1	Boolean
6	BP	Patient has blood pressure	0,1	Boolean
7	Anaemia	Red blood cell deficiency	0,1	Boolean
8	Age	Patient age	40–49	Years
9	Sodium	Sodium level in body	114–148	mEq/L
10	Creatinine	Creatinine level in body	0.50–9.40	mg/dL
11	Platelets	Blood platelets	25.01–850	Kiloplatelets/mL

**Table 5:** Parametric values for CNN.

Parameter	Value
Embedding dimension	300
Pooling	2 × 2
Optimizer	Adam
No. of filters	5 × 64
Epochs	25
Max_Sequence_length	11
Function	Binary cross entropy
Batch size	256

**Table 6:** System specifications.

Sr. No.	Hardware	Software
1	RAM 8 GB	Windows 10
2	DDR4	Anaconda 1.0.0
3	Core i-5	Pycharm 2023.1.1
4	6th Generation	Python 3.7.3

**Table 7:** Performance of deep learning models [59].

Model	*Accuracy*	*Precision*	*Recall*	F1Score
CNN	0.9398	0.95	0.95	0.95
MLP	0.9120	0.94	0.94	0.94
RNN	0.9100	0.89	0.91	0.90
LSTM	0.9691	0.93	0.93	0.93
Random Forest (RF) without SMOTE [59]	0.8889	0.89	0.89	0.89
ETC with SMOTE [59]	0.9262	0.93	0.93	0.93

**Table 8:** Accuracy comparison of machine learning and deep learning models.

Models	Accuracy
DT [59]	0.8778
AdaBoost [59]	0.8852
LR [59]	0.8442
SGD [59]	0.5491
RF [59]	0.9188
GBM [59]	0.8852
ETC [59]	0.9262
GNB [59]	0.7540
SVM [59]	0.7622
RNN	0.9100
LSTM	0.9691
MLP	0.9120
CNN	0.9398

**Table 9:** Comparison of transfer learning models with the proposed CNN.

Model	*Accuracy*	*Precision*	*Recall*	F1Score
VGG-16	0.9291	0.89	0.90	0.90
AlexNet	0.9170	0.89	0.89	0.89
CNN	0.9398	0.95	0.95	0.95

**Table 10:** Training time of classifiers.

Model	Training Time
Proposed approach	24 min
VGG-16	29 min
AlexNet	32 min

**Table 11:** Results for 10-fold cross-validation of CNN model.

Fold Number	*Accuracy*	*Precision*	*Recall*	*F1-Score*
F1	0.951	0.961	0.912	0.922
F2	0.921	0.970	0.934	0.962
F3	0.931	0.932	0.932	0.943
F4	0.981	0.970	0.994	0.953
F5	0.940	0.934	0.984	0.916
F6	0.961	0.962	0.974	0.923
F7	0.942	0.970	0.961	0.914
F8	0.941	0.941	0.954	0.973
F9	0.920	0.942	0.945	0.981
F10	0.974	0.954	0.975	0.994
**Average**	**0.9462**	**0.9398**	**0.9565**	**0.9481**

**Table 12:** Comparison of the proposed system with existing systems. ✓ indicates the existence of feature while × indicates the non-existence of particular feature.

Reference	Features > 5	Patient Monthly Record	AI Technique	IoT
[60]	×	×	✓	✓
[61]	×	×	✓	✓
[62]	×	×	✓	✓
[63]	×	×	✓	✓
[64]	×	×	✓	✓
[65]	×	×	×	✓
[66]	×	×	×	✓
[67]	×	×	×	✓
**Proposed Model**	✓	✓	✓	✓

**Table 13:** Performance comparison of the proposed model with state-of-the-art approaches.

Authors	Models	Accuracy
Kumar Dwivedi [68]	Logistic regression	0.85
Parthiban et al. [69]	Naïve Bayes	0.74
Shah et al. [70]	K-NN	0.90
Vembandasamy et al. [71]	Naïve Bayes	0.86
**Proposed Model**	**CNN**	**0.9398**

**Table 14:** Real-time cardiovascular disease prediction dataset.

Feature	Attribute	Domain	Data Type	Mean	STD	Missing Values (%)
Age	age	Age in years: 29–77	Real	54	9	0.00
Sex	sex	Male = 1, Female = 0	Binary			0.00
Chest pain type	cp	1 = typical angina	Nominal			0.00
		2 = atypical angina				
		3 = non-anginal pain				
		4 = asymptomatic				
Resting Blood Pressure in mm/Hg	trestbps	94–200	Real	131.344	17.862	6.41
Serum Cholesterol in mg/dL	chol	126–564	Real	249.659	51.686	3.26
Fasting blood sugar > 120 mg/dL	fbs	1 = yes, 0 = no	Binary			9.78
Resting ECG observations	restecg	0 = normal	Nominal			0.22
		1 = having ST-T wave abnormality (T wave inversions and/or ST elevation or depression of >0.05 mV)				
		2 = showing probable or definite left ventricular hypertrophy by Estes’ criteria				
Maximum heart rate achieved	thalach	71–202	Real	149.678	23.166	5.98
Exercise-induced angina	exang	1 = yes, 0 = no	Binary			5.98
ST depression induced by angina relative to rest	oldpeak	0–6.2	Real	1.05	1.145	6.74
Slope of the peak exercise ST segment	slope	1 = upsloping	Ordered			33.58
		2 = flat				
		3 = downsloping				
Number of major vessels colored by fluoroscopy	ca	Number of vessels: 0, 1, 2, 3	Real			66.43
Thallium stress test result	thal	3 = normal; 6 = fixed defect	Nominal			52.83

**Table 15:** Performance of deep learning models using the real-time dataset.

Model	*Accuracy*	*Precision*	*Recall*	F1Score
CNN	0.9534	0.93	0.97	0.95
MLP	0.9302	0.91	0.93	0.92
RNN	0.9449	0.92	0.92	0.92
LSTM	0.9329	0.91	0.92	0.91

**Table 16:** Comparison with existing approaches from [73].

Stacking Classifiers	*Precision*	*Recall*	F1Score
KNN, XGB, ADA [73]	0.888	0.890	0.889
KNN, XBG, SVM [73]	0.878	0.878	0.878
KNN, XGB, MLPC [73]	0.910	0.910	0.910
KNN, XGB, MLPC, ADA [73]	0.871	0.875	0.873
XGB, MLPC, ADA, SVM [73]	0.882	0.886	0.884
KNN, XGB, MLPC, ADA, SVM [73]	0.872	0.873	0.872
**Proposed CNN**	0.934	0.975	0.956

## Data Availability

The dataset and code are available from the corresponding author on reasonable request.
